# Characteristics and Outcomes of COVID-19 Patients Admitted to Intensive Care Units in a Large Health System in Western Pennsylvania

**DOI:** 10.7759/cureus.16552

**Published:** 2021-07-22

**Authors:** Adeel Nasrullah, Thejus Jayakrishnan, Patrick Wedgeworth, Melissa Mosley, Kirtivardhan Vashistha, Aaron Haag, Veli Bakalov, Abhishek Chaturvedi, Susan Manzi, Anastasios Kapetanos

**Affiliations:** 1 Pulmonary and Critical Care Medicine, Allegheny Health Network, Pittsburgh, USA; 2 Internal Medicine, Allegheny Health Network, Pittsburgh, USA; 3 Rheumatology, Allegheny Health Network, Pittsburgh, USA

**Keywords:** covid-19, sars-cov-2, viral pneumonia, pandemic, icu, mortality

## Abstract

Importance

Despite growing literature, there is still limited understanding of factors that can predict outcomes in coronavirus disease 2019 (COVID-19) patients who require intensive care.

Objective

To evaluate the characteristics of COVID-19 patients admitted to the intensive care unit (ICU) and identify their associations with outcomes.

Background

There are limited data on the outcomes in COVID-19 patients in Pennsylvania.

Design

Retrospective study

Setting

Intensive care units in an academic health system in Western Pennsylvania.

Participants

Patients with reverse transcriptase-polymerase chain reaction (RT-PCR)-confirmed COVID-19 admitted to ICUs as direct admission or transfers from regular floors between March 1, 2020, and April 30, 2020.

Main outcome(s) and measure(s)

The primary outcome was inpatient mortality. Secondary outcomes included complications during ICU stay, hospital length of stay, discharge disposition, and the need for oxygen at discharge. Categorical variables are described as frequencies and continuous variables as median with interquartile range (IQR). Regression modeling was used to identify the predictors of inpatient mortality in these patients. P-value <0.05 was considered statistically significant. Analysis was performed using Stata version 15.1 (StataCorp, College Station, Texas).

Results

The cohort included 58 consecutive patients, with a median age of 62 years (IQR 54-73), 63.8% of which were male. On presentation, constitutional symptoms were the most common (91.4%), followed by lower respiratory tract symptoms (87.9%). Tachypnea (65.5%) and hypoxia (67.2%) were the most common abnormal vital signs at presentation. Common comorbidities were cardiovascular disease (74.1%), obesity (53.5%), and diabetes (39.7%). The median Acute Physiology and Chronic Health Evaluation (APACHE) score on admission to ICU was 11 (IQR 8.5-17.5). The major complications included acute respiratory distress syndrome (ARDS) 50.0%, shock 41.4%, and acute kidney injury 41.4%. The proportion of patients who underwent mechanical ventilation, required vasopressors, or were on renal replacement therapy were 58.6%, 41.4%, and 10.3%, respectively. Overall mortality was 32.8%. Age, Charlson-comorbidity index, tachypnea, lymphopenia at presentation, high APACHE score, shock, ARDS, mechanical ventilation, and steroid use were significantly associated with mortality. Of the patients who survived their ICU stay, 63.2% were discharged home and 44.7% had a new oxygen requirement at discharge.

Conclusion and relevance

Our study reports high mortality in COVID-19 patients requiring ICU care in Western Pennsylvania. Identifying factors associated with poor prognosis could help risk-stratify these patients. Prospective studies are needed to assess whether early risk stratification and triaging result in improved outcomes.

## Introduction

Since its emergence, coronavirus disease 2019 (COVID-19) has remained a global health crisis and continues to impart significant social, psychological, and logistical burdens on individuals and health care systems [1-2]. The United States has reported the largest number of cases, and as of July 13, 2021, there were 33,726,363 COVID-19 cases with 605,140 deaths [3]. Robust research has resulted in a better understanding of the natural history of the disease, characteristics of patients, and predictors of outcomes. Despite this, there is still a need for more evidence data regarding characteristics and outcomes of those admitted to intensive care units (ICUs), particularly in the United States. Preliminary studies suggest that up to 6% of COVID-9 patients in the US required ICU admission, conferring the highest mortality to this group [4].

Allegheny Health Network (AHN) is one of the largest consortiums of academic urban and non-academic rural hospitals in Western Pennsylvania and has been at the forefront of COVID-19 in Pennsylvania [5-7]. Up to July 13, 2021, the state of Pennsylvania reported 1,214,654 COVD-19 cases with 27,769 deaths, however, characteristics specific to severely ill patients have not been published [8]. In this context; the present study was conducted to describe the initial experience with regards to the clinical characteristics and outcomes for patients with COVID-19 admitted to ICUs across a large health system. Factors associated with mortality in this population were also identified.

## Materials and methods

A retrospective study was conducted of consecutive patients diagnosed with a COVID-19 by real-time polymerase chain reaction (RT-PCR) test via nasal swab admitted to intensive care units (ICUs) between March 1, 2020, and April 30, 2020. The cohort included patients transferred to ICU for escalation of care and direct admissions from the emergency department. For patients with readmissions, only index hospitalization due to COVID-19 is included in this study. Patient disposition to the ICU was determined by the need for mechanical ventilation, vasopressors, or the clinical judgment of providers. The severity of illness was determined by sequential organ assessment failure (SOFA) and the acute physiology and chronic health evaluation 2 (APACHE 2) scoring system.

The AHN Institutional Review Board approved the study and waived the need for informed consent. Deidentified data collection was performed from the electronic health record system (Epic) and included patient demographics, medical history, home medications, clinical presentation characteristics, and treatment strategies. The symptoms were grouped into the following categories: constitutional (anorexia, fever, chills, myalgia or arthralgia, and fatigue), upper respiratory tract (sore throat, nasal congestion, anosmia, and dysgeusia), lower respiratory tract (cough, dyspnea, sputum, hemoptysis), and others (chest pain, palpitation, lightheadedness, syncope, abdominal pain, diarrhea, nausea or vomiting, conjunctival injection, altered mental status, headache, and muscle weakness). Headache was included in the “others” category, as it could be both a constitutional and a neurological symptom. Comorbidities were classified as follows: cardiovascular (congestive heart failure, coronary artery disease, arrhythmias, valvular disease, hypertension, pulmonary hypertension, peripheral vascular disease, other cardiac diseases), pulmonary (chronic obstructive pulmonary disease, asthma, interstitial lung disease, pulmonary hypertension, other lung diseases), renal disease (chronic kidney disease, including end-stage renal disease requiring dialysis). Charlson-comorbidity index (CCI) was also calculated for analysis.

The primary outcome was mortality during the hospital stay. Secondary outcomes included complications during the ICU stay (acute respiratory distress syndrome (ARDS), shock, acute kidney injury, deep venous thrombosis (DVT) or pulmonary embolism (PE), bleeding, and cardiac arrest), hospital length of stay, discharge disposition (home versus assisted care facility), and oxygen need at discharge. ARDS was defined using the Berlin Criteria (presence of acute respiratory failure with bilateral pulmonary infiltrates, a ratio of arterial oxygen tension to a fraction of inspired oxygen <300 with >5 cm water of positive-end expiratory pressure, and absence of cardiogenic pulmonary edema) [9]. Acute kidney injury was defined as an absolute increase in serum creatinine of more than or equal to 0.3 mg/dl, a percentage increase in serum creatinine of more than or equal to 50% (1.5-fold from baseline), or a reduction in urine output (documented oliguria of less than 0.5 ml/kg per hour for more than six hours) [10].

Categorical outcomes are described in percentages and continuous variables with median and interquartile range (IQR). Univariate logistic regression analysis for mortality was performed for variables with at least 20 observations. Multivariate regression was not performed due to the small sample size and established interactions among different variables resulting in a model that may not add meaningful additional information to the existing results. Results are represented as odds ratio (OR) with a 95% confidence interval (95% CI) for applicable variables. Statistical tests were two-tailed, and statistical significance was defined as p-value < .05. Analyses were performed using Stata version 15.1 (StataCorp, College Station, Texas). Since the analyses have not been adjusted for multiple comparisons and is prone to type-I error, the findings should be interpreted as exploratory.

## Results

Characteristics of patients admitted to ICU

Patient characteristics are outlined in Table 1. The cohort included 58 patients, with a median age of 62 years (IQR 54-73), and included 37 (63.8%) males. The majority of patients were non-Hispanic White (60.3%) followed by non-Hispanic Black (29.3%). Constitutional symptoms were reported by 91.4% while 87.9% reported lower respiratory tract symptoms, and 20.7% reported upper respiratory symptoms. Common comorbidities included cardiovascular disease (74.1%), obesity (53.5%), and diabetes (39.7%). The median CCI was 1 (IQR 0-2).

**Table 1:** Characteristics for patients admitted with COVID-19 to the ICU ^a ^Continuous variables are reported as median (IQR) and categorical variables as frequency (percentage). IQR=Interquartile Range; HCP=Healthcare Provider; BMI=Body Mass Index; ACE=Angiotensin-Converting Enzyme; ARB=Angiotensin Receptor Blocker; CXR=Chest X-Ray; AST=Aspartate Aminotransferase; ALT=Alanine Transaminase; APACHE 2=Acute Physiology and Chronic Health Evaluation 2; SOFA=Sequential Organ Assessment Failure

Characteristics	Value ^a^
Age (in years)	62 (54-73) years
Male	37 (63.8%)
Race	
Non-Hispanic White	35 (60.3%)
Non-Hispanic Black	17 (29.3%)
Hispanic	2 (3.5%)
Other/Unknown	4 (6.9%)
Symptoms	
Constitutional	53 (91.4%)
Lower respiratory symptoms	51 (87.9%)
Upper respiratory symptoms	12 (20.7%)
Other systems/nonspecific symptoms	38 (65.5%)
Comorbidities	
Cardiovascular	43 (74.1%)
Obesity (BMI>30kg/m^2^)	31 (53.5%)
Diabetes	23 (39.7%)
Pulmonary	9 (15.5%)
Renal	8 (13.8%)
Other	23 (39.7%)
ACE/ARB	22 (37.9%)
Smoking	
Active smoker	23 (39.7%)
Admission Vitals	
Temperature>38 degree-Celsius	17 (29.3%)
Heart rate>100 beats per minute	27 (46.6%)
Respiratory rate>20 breaths per minute	38 (65.5%)
Systolic blood pressure (mm Hg) < 90	1 (1.7%)
Hypoxia at presentation	39 (67.2%)
Abnormal CXR (n=52)	44 (84.6%)
Hospital admission Abnormal Lab Findings	
AST/ALT>40 U/L	36 (62.1%)
Absolute lymphocyte count<0.60k/mcl	17 (29.3%)
Hospital admission labs	
White blood cell count k/mcl	6.8 (5.8-10.5)
Creatinine mg/dl	1.1 (0.8-1.5)
Procalcitonin ng/ml (n=44)	0.2 (0.1-0.4)
Lactate dehydrogenase U/l (n=34)	503 (377-778)
D-dimer mg/ml (n=31)	1.5 (1.1-3.8)
Pro-brain natriuretic peptide pg/ml (n=29)	291 (131-2557)
C-reactive protein mg/dl (n=28)	11.9 (6.4-17.4)
Erythrocyte sedimentation rate mm/hour (n=6)	51 (29-84)
ICU Admission severity of illness	
APACHE 2 (40)	11 (8.5-17.5)
SOFA (44)	3 (2-5)
Treatment Strategies received	
Hydroxychloroquine	45 (77.6%)
Empiric antibiotics	44 (75.9%)
Mechanical ventilation	34 (58.6%)
Vasopressor use	24 (41.4%)
Steroids	20 (34.5%)
Neuromuscular blockade use	11 (19.0%)
Proning	10 (37.9%)
Blood transfusion	9 (15.5%)
Renal replacement therapy	5 (8.6%)
Plasmapheresis	4 (6.9%)
Intravenous immunoglobulin	3 (5.2%)
Mechanical circulatory support	1 (1.7%)

Upon presentation, 65.5% patients were tachypneic, 67.2% were hypoxic, and 46.6% were tachycardic. Important admission laboratory abnormalities included transaminitis in 62.1% of patients, lymphopenia in 29.3%, and abnormal chest X-ray in 84.6% of patients among those who had the test performed.

The most common abnormal chest X-ray finding was interstitial opacities in 86.3% (38 of 44 patients). Fourteen patients had admission chest computed tomography (CT) and were 100% abnormal. Inflammatory markers (C-reactive protein (CRP), erythrocyte sedimentation rate (ESR), D-dimer) were not obtained in all patients but were elevated in those measured. The median APACHE 2 score at admission to ICU was 11 (IQR 8.5-17.5), and the sequential assessment of organ failure score was 3 (IQR 2-5). Table 1 summarizes the characteristics of patients admitted to ICU.

ICU treatments

Following admission to the ICU, 58.6% required mechanical ventilation, 41.4% required vasopressor support, 8.6% needed renal replacement therapy, and one patient needed mechanical circulatory support (Table 1). Pharmacological treatment strategies included hydroxychloroquine in 77.6%, empiric antibiotics in 75.9%, steroids in 34.5%, plasmapheresis in 6.9%, and intravenous immunoglobulin in 5.2% of patients.

ICU outcomes and predictors of inpatient mortality

Overall, inpatient mortality was 32.8% in our cohort. The major complications included: ARDS in 50.0%, shock in 41.4%, AKI in 41.4%, venous thromboembolism (DVT/PE) in 7.0%, and bleeding in 6.9% patients. The median total length of stay for this cohort was 11.5 days (IQR 5-20). Median length of stay in the ICU was six days (IQR 3-16) to 13 days (4-21) for survivors versus five days (IQR 2-11) for non-survivors. Among survivors, 63.2% were discharged home and 34.2% to assisted care facility. Additionally, 44.7% patients had a new oxygen requirement upon discharge. On univariate regression modeling, age [OR 1.05(1.01-1.11) p-value=0.015], CCI [OR 1.37(1.02-1.82) p-value=0.032], tachypnea [OR 17.1(2.1-141.0) p-value=0.008] and lymphopenia [OR 3.9(1.2-13.0) p-value=0.028] on admission, APACHE-2 score [OR 1.13(1.03-1.24) p-value=0.002], steroid use [OR 4.6(1.4-14.9)p-value=0.010], development of ARDS [OR 4.5(1.3-15.0) p-value=0.015], and shock [OR 8.1 (2.3-28.3) p=0.004] were found to be associated with inpatient mortality. ICU outcomes and predictors of inpatient mortality are summarized in Tables 2-3.

**Table 2:** Outcomes for patients admitted to ICU ^a ^Continuous variables are reported as median (IQR) and categorical variables as frequency (percentage). IQR=Interquartile Range; HCP=Healthcare Provider; ARDS=Acute Respiratory Distress Syndrome; DVT=Deep Venous Thrombosis; PE=Pulmonary Embolism b Alive at discharge

Outcomes	Value ^a^
Mortality	19 (32.8%)
Complications	
ARDS	29 (50.0%)
Shock	24 (41.4%)
Acute kidney injury	24 (41.4%)
DVT/PE	4 (6.9%)
Bleeding	4 (6.9%)
Cardiac arrest	3 (5.2%)
Hospital length of stay, days	11.5 (5-20)
Discharge disposition (n=38)^b^	
Home	24 (63.2%)
Assisted care facility	13 (34.2%)
Outside hospital	1 (2.6%)
New oxygen at discharge	17 (44.7%)

**Table 3:** Significant factors associated with mortality of patients admitted to ICU ^a ^Continuous variables are reported as median (IQR) and categorical variables as frequency (percentage). IQR=Interquartile Range; HCP=Healthcare Provider; CCI=Charlson-Comorbidity Index; BMI=Body Mass Index; ACE=Angiotensin-Converting Enzyme; ARB=Angiotensin Receptor Blocker; CXR=Chest X-Ray; AST=Aspartate Aminotransferase; ALT=Alanine Transaminase; APACHE 2=Acute Physiology and Chronic Health Evaluation 2; HCQ=Hydroxychloroquine

Characteristics	Survivors (n=39) Value ^a^	Non-Survivors (n=19) Value ^a^	Odds ratio (95% CI)	P-value
Age (in years)	61 ((54-67)	65 (60-79)	1.05 (1.01-1.11)	0.015
Male	23 (59.0%)	14 (73.7%)	-	0.26
Race				
Non-Hispanic White	23 (59.0%)	12 (63.2%)	Reference	
Non-Hispanic Black	11 (28.2%)	6 (31.6%)	-	0.94
Hispanic	2 (5.1%)	0	Not performed	
Other/Unknown	3 (7.7%)	1 (5.3%)	-	0.71
Symptoms				
Constitutional	38 (97.4%)	15 (79.0%)	0.10 (0.01-0.95))	0.02
Lower respiratory symptoms	12 (30.8%)	17 (89.5%)		0.80
Upper respiratory symptoms	34 (87.2%)	0	Not performed	
Other systems/nonspecific symptoms	26 (66.7%)	12 (63.2%)		
Comorbidities				
Cardiovascular	27 (69.2%)	16 (84.2%)	-	0.21
Obesity (BMI>30 kg/m^2^)	22 (56.4%)	9 (47.4%)	-	0.52
Diabetes	15 (38.5%)	8 (42.1%)	-	0.79
Pulmonary	6 (15.4%)	3 (15.8%)	-	0.96
Renal	4 (10.3%)	4 (21.1%)	-	0.28
Other	13 (33.3%)	10 (52.6%)	-	0.16
CCI	1 (0-1)	1 (0-4)	1.37 (1.02-1.82)	0.032
ACE/ARB	24 (61.5%)	6 (31.6%)	-	0.48
Smoking				
Active smoker	16 (41.0%)	7 (36.8%)	-	0.80
Admission Vitals				
Temperature>38 degree-Celsius	11 (28.2%)	6 (31.6%)	-	0.79
Heart rate>100 beats per minute	16 (41.0%)	11 (57.9%)	-	0.22
Respiratory rate>20 breaths per minute	20 (51.3%)	18 (94.7%)	17.1 (2.1-141.0)	0.008
Systolic blood pressure (mm Hg) < 90	0	1 (5.3%)	Not performed	
Hypoxia at presentation	24 (61.5%)	15 (79.0%)	-	0.17
Abnormal CXR	30 (83.3%)	14 (87.5%)		0.70
Hospital admission Abnormal Lab Findings				
AST/ALT>40 U/L	22 (56.4%)	14 (73.7%)	2.2 (0.7-7.2)	0.20
Absolute lymphocyte count<0.60k/mcl (n=57)	8 (20.5%)	9 (50%)	3.9 (1.2-13.0)	0.028
Hospital admission labs		.		
White blood cell count k/mcl	7.0 (5.8—10.0)	6.7 (5.0-12.5)	-	0.50
Creatinine mg/dl	1.0 (0.8-1.3)	1.5 (0.9-2.0)	-	0.07
Lactate dehydrogenase U/l (n=34)	600 (353-845)	488 (395-687)	-	0.45
D-dimer mg/ml (n=31)	1.3 (1.0-2.1)	2.4 (1.5-5.2)	-	0.13
Pro-brain natriuretic peptide pg/ml (n=29)	219 (67-408)	2152 (297.5-6202.5)	-	0.08
C-reactive protein mg/dl (n=28)	10.4 (6.2-13.6)	16.7 (10.5-26.7)	-	0.13
Erythrocyte Sedimentation Rate mm/hr (n=6)	29.0 (9-42)	84 (60-123)	Not performed	
ICU admission severity of illness				
APACHE 2 (40)	10 (7-11)	16 (11-25)	1.13 (1.03-1.24)	0.002
Treatment strategies received				
Mechanical ventilation	16 (41.0%)	18 (94.7%)	25.9 (3.1-213.9)	<0.0005
HCQ	30 (76.9%)	15 (79.0%)	-	0.86
Empiric antibiotics	28 (71.8%)	16 (84.2%)	-	0.29
Pressors	10 (25.6%)	14 (73.7%)	8.1 (2.3-28.3)	0.004
Steroids	9 (23.1%)	11 (57.9%)	4.6 (1.4-14.9)	0.010
Neuromuscular blockade use	7 (18.4%)	4 (22.2%)	Not performed	
Proning	11 (28.2%)	11 (67.9%)	1.0 (0.9-1.2)	0.41
Blood transfusion	3 (7.7%)	6 (31.6%)	Not performed	
Renal replacement therapy	2 (5.3%)	3 (15.8%)	Not performed	
Plasmapheresis	1 (2.6%)	3 (15.8%)	Not performed	
Intravenous immunoglobulin	2 (5.1%)	1 (5.3%)	Not performed	
Mechanical circulatory support	1 (2.6%)	18 (94.7%)	Not performed	
Complications				
ARDS	15 (38.5%)	14 (73.7%)	4.5 (1.3-15.0)	0.015
Shock	10 (25.6%)	14 (73.7%)	8.1 (2.3-28.3)	0.0004
Acute kidney injury	13 (33.3%)	11 (57.9%)	2.8 (0.9-8.5)	0.08
DVT/PE	2 (5.1%)	0	Not performed	
Bleeding	0	4 (21.1%)	Not performed	
Cardiac arrest	0	3 (15.8%)	Not performed	

## Discussion

In this retrospective study of 58 COVID-19 patients admitted to the ICU, our findings are consistent with prior evidence from institutions within and outside of the United States as described below.

The cohort was predominantly male and older, similar to other studies [11-15]. The majority of patients had constitutional and lower respiratory tract symptoms at presentation, and more than 60% had respiratory distress at presentation [11-12,14,16-17]. Similarly, the cohort had abnormally elevated levels of inflammatory markers (D-dimer, CRP, ESR) at presentation. A definitive comparison of biomarkers in terms of risks for ICU admission or mortality cannot be made, as it was recognized late in the study period that admission and daily levels of these biomarkers were warranted in all patients [13]. Future studies have reported that older patients, the presence of type-2 diabetes, cancer, in-hospital complications (such as acute kidney injury, sepsis/septic shock, multiorgan dysfunction), and higher D-dimer > 5,000 ng/mL are associated with higher mortality. Although the prevalence of lymphocytopenia is lower than previously reported (63%-70%), it is associated with increased mortality [11,13,16-19]. Our study also found a statistically significant relationship between tachypnea with mortality. These factors should be considered when risk-stratifying patients.

A higher rate of comorbidities (58-86%) has been reported in this patient group [12,15,19-20]. Common comorbidities include cardiovascular disease, obesity, and diabetes. Early studies from China, Italy, and New York on COVID-19 patients have shown an association of case fatality with medical comorbidities, and investigators from Italy demonstrated the potential relationship of comorbidities such as hypertension with mechanical ventilation parameters [12,15,19-20]. In the current study, comorbidities were not associated with mortality, however, the cumulative effect of comorbidities in terms of CCI and age were associated with increased mortality [17,20-21]. The lack of direct correlation between types of comorbidities and outcomes could present a type-1 error, again, due to the small sample size of this study.

Once admitted to the ICU, patients suffered a multitude of complications, including ARDS, shock, and renal failure, which increased the risk for mortality similar to other studies [18,21]. The mortality in the present study was 33%. The rates for critically ill COVID-19 patients from other centers are variable, as follows in descending order: 67% (Seattle, Arentz et al.), 62% (China, Yang et al.), 50% (Seattle, Bhatraju et al.), 40% (New York, Cummings et al.), 39% (Italy, Grasselli et al.), 39% (China, Wang et al.), 38% (China, Huang et al.), 31% (Atlanta, Sara et al.), and 14% (California, Ferguson et al.) [11-12,14-18,21-22]. The association of mechanical ventilation, the need for vasopressors, and renal replacement therapy with mortality may simply indicate the effect of the multiorgan failure but also suggests that despite aggressive management, there is a high risk for treatment failure leading to death. Other studies from the US and Italy also indicate a high incidence of ARDS and a high rate of mechanical ventilation (60%-88%) [1,11-12,14-15,21,22]. Mechanical ventilation rates were lower in early studies from China: 15% (Huang et al.), 42% (Yang et al.), and 47% (Wang et al.) [13,16,18]. This variability could represent the differences in population characteristics, as well as the provision of care, including the use of non-invasive ventilation strategies. There is also a possibility that analyses in several of the above-cited studies included patients who were still admitted to the hospital at the time of the study, resulting in underestimation of mortality and morbidity. Nearly half of patients required oxygen at discharge, however, the long-term need for oxygen is unknown, as is the ultimate impact on pulmonary physiology. Specific aspects of treatment such as strategies used for mechanical ventilation, types, and doses of vasopressors are beyond the scope of this study.

Lastly, since this study represents patients admitted during the earlier phases of COVID-19, treatment strategies did not include remdesivir, interleukin 6 (IL-6) antagonists, convalescent plasma, or dexamethasone, which are now commonly utilized [23]. The higher rate of mortality among those requiring steroids was likely a surrogate for the severity of illness for these patients and is similar to observational studies in pneumonia caused by COVID-19, as well as other phylogenetically similar viruses [14,19]. Hydroxychloroquine was used for the majority of patients, and at least in unadjusted comparison, did not appear to impact survival. Another aspect to be explored is the use of non-invasive ventilation (NIV). In an Italian study of critically ill COVID-19 patients, 11% with acute hypoxemic respiratory failure were managed with NIV [11]. The use and impact of NIV on outcomes were not included in this study.

The limitations of the present study include those inherent to retrospective study design such as recall biases, indication biases, and the presence of uncharacterized confounding factors. While a multivariate regression analysis could have helped identify how various factors impacted mortality in a combined model, it was not performed due to the complexity of the resultant model, which failed to significantly add to the presented results. We plan to perform this in the future with a larger dataset. The follow-up was limited to the inpatient hospital stay and did not capture complications or readmission post-discharge. The data included are from eight different hospitals with a mix of urban academic and rural community hospitals. Variations may exist due to the practice and population patterns of the individual hospitals.

## Conclusions

The present study contributes to the growing body of data on the characteristics and outcomes for COVID-19 patients admitted to the ICU. COVID-19 was associated with high mortality and complication rates. Several factors associated with adverse outcomes were identified in the present study. Further investigations are needed to assess the predictive factors that drive mortality to better refine treatment strategies.

## References

[1] Richardson S, Hirsch JS, Narasimhan M (2020). Presenting characteristics, comorbidities, and outcomes among 5700 patients hospitalized with COVID-19 in the New York City Area. JAMA.

[2] Desai HD, Sharma K, Jadeja DM, Desai HM, Moliya P (2020). COVID-19 pandemic induced stress cardiomyopathy: a literature review. Int J Cardiol Heart Vasc.

[3] (2020). CDC. Coronavirus disease 2019 (COVID-19) in the U.S. https://www.cdc.gov/coronavirus/2019-ncov/cases-updates/cases-in-us.html.

[4] (2020). CDC. Coronavirus disease 2019 (COVID-19). https://www.cdc.gov/coronavirus/2019-ncov/need-extra-precautions/racial-ethnic-minorities.html.

[5] Allegheny Health Network (2020). Wikipedia. Allegheny Health Network. https://en.wikipedia.org/wiki/Allegheny_Health_Network.

[6] (2020). Coronavirus response. Allegheny Health Network. https://www.ahn.org/coronavirus.html.

[7] Boden S (2020). Allegheny Health Network starts drive-through testing for COVID-19. https://www.wesa.fm/science-health-tech/2020-03-18/allegheny-health-network-starts-drive-through-testing-for-covid-19.

[8] (2020). Pennsylvania COVID-19 numbers. Department of Health. https://www.health.pa.gov/topics/disease/coronavirus/Pages/Cases.aspx.

[9] Ranieri VM, Rubenfeld GD, Thompson BT (2012). Acute respiratory distress syndrome: the Berlin Definition. JAMA.

[10] Mehta RL, Kellum JA, Shah SV, Molitoris BA, Ronco C, Warnock DG, Levin A (2007). Acute Kidney Injury Network: report of an initiative to improve outcomes in acute kidney injury. Crit Care.

[11] Arentz M, Yim E, Klaff L, Lokhandwala S, Riedo FX, Chong M, Lee M (2020). Characteristics and outcomes of 21 critically ill patients with COVID-19 in Washington State. JAMA.

[12] Grasselli G, Zangrillo A, Zanella A (2020). Baseline characteristics and outcomes of 1591 patients infected with SARS-CoV-2 admitted to ICUs of the Lombardy Region, Italy. JAMA.

[13] Wang D, Hu B, Hu C (2020). Clinical characteristics of 138 hospitalized patients with 2019 novel coronavirus-infected pneumonia in Wuhan, China. JAMA.

[14] Bhatraju PK, Ghassemieh BJ, Nichols M (2020). Covid-19 in critically ill patients in the Seattle region - case series. N Engl J Med.

[15] Cummings MJ, Baldwin MR, Abrams D (2020). Epidemiology, clinical course, and outcomes of critically ill adults with COVID-19 in New York City: a prospective cohort study. Lancet.

[16] Huang C, Wang Y, Li X (2020). Clinical features of patients infected with 2019 novel coronavirus in Wuhan, China. Lancet.

[17] Wang Y, Lu X, Li Y (2020). Clinical course and outcomes of 344 intensive care patients with COVID-19. Am J Respir Crit Care Med.

[18] Yang X, Yu Y, Xu J (2020). Clinical course and outcomes of critically ill patients with SARS-CoV-2 pneumonia in Wuhan, China: a single-centered, retrospective, observational study. Lancet Respir Med.

[19] Zhou F, Yu T, Du R (2020). Clinical course and risk factors for mortality of adult inpatients with COVID-19 in Wuhan, China: a retrospective cohort study. Lancet.

[20] (2020). Characteristics of and Important Lessons From the Coronavirus Disease 2019 (COVID-19) Outbreak in China: Summary of a Report of 72 314 Cases From the Chinese Center for Disease Control and Prevention | Global Health | JAMA | JAMA Network. Accessed July 3. https://jamanetwork.com/journals/jama/fullarticle/2762130.

[21] Auld SC, Caridi-Scheible M, Blum JM (2020). ICU and ventilator mortality among critically ill adults with coronavirus disease 2019. Crit Care Med.

[22] Ferguson J, Rosser JI, Quintero O (2020). Characteristics and outcomes of coronavirus disease patients under nonsurge conditions, Northern California, USA, March-April 2020. Emerg Infect Dis.

[23] (2020). NIH. What’s new. Coronavirus disease COVID-19. COVID-19 treatment guidelines. https://www.covid19treatmentguidelines.nih.gov/whats-new/.

